# Droplet Memory on Liquid-Infused Surfaces

**DOI:** 10.1021/acs.langmuir.3c00289

**Published:** 2023-04-17

**Authors:** Davide Bottone, Stefan Seeger

**Affiliations:** Department of Chemistry, University of Zurich, Winterthurerstrasse 190, 8057 Zurich, Switzerland

## Abstract

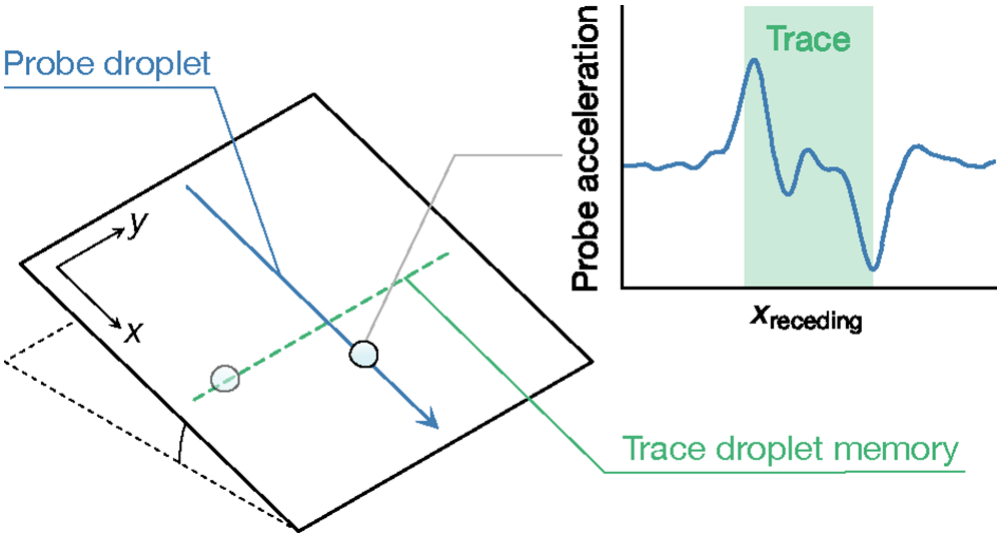

The knowledge of droplet friction on liquid-infused surfaces (LIS) is of paramount importance for applications involving liquid manipulation. While the possible dissipation mechanisms are well-understood, the effect of surface texture has thus far been mainly investigated on LIS with highly regular solid topographies. In this work, we aim to address this experimental gap by studying the friction experienced by water droplets on LIS based on both random and regular polysilsesquioxane nanostructures. We show that the available models apply to the tested surfaces, but we observe a previously unreported droplet memory effect: as consecutive droplets travel along the same path, their velocity increases up to a plateau value before returning to the original state after a sufficiently long time. We study the features of this phenomenon by evaluating the motion of droplets when they cross the path of a previous sequence of droplets, discovering that moving droplets create a low-friction trace in their wake, whose size matches their base diameter. Finally, we attribute this to the temporary smoothing out of an initially conformal lubricant layer by means of a Landau–Levich–Derjaguin liquid film deposition behind the moving droplet. The proposed mechanism might apply to any LIS with a conformal lubricant layer.

## Introduction

Since their inception a decade ago,^1,2^ liquid-infused surfaces (LIS) have attracted considerable attention in academia and industry alike, owing to their remarkable liquid repellency, resistance to fouling, and self-healing behavior.^3−5^

One particularly promising application of these surfaces, among many notable applications, is their use for precise droplet manipulation.^6^ As a matter of fact, droplet mobility on LIS can be tuned with an appropriate selection of surface topography and lubricant properties,^7−9^ and the direction of motion can be controlled with the introduction of surface anisotropy and texture gradients.^10−14^

It is therefore clear that a fundamental understanding of droplet friction on LIS is key to a robust design of their properties for this application. In this context, water droplets moving on a LIS infused with a viscous lubricant oil, such that η_o_ ≫ η_w_, where η_o_ and η_w_ are the viscosities of oil and water, respectively, is by far the most common scenario. In this limiting case, viscous dissipation and lubricant depletion are mainly localized in the annular wetting ridge surrounding the droplet^9,15−17^ and arise from a combination of Landau–Levich–Derjaguin (LLD) thin-film deposition at the receding menisci^18,19^ and interfacial shear stresses on the texture asperities at the advancing menisci.^8,9^

While the friction behavior captured by this model is supported by ample experimental evidence, the effect of surface texture has only been investigated for rigid, highly regular lithographed surfaces.^8,9^ This might pose a limitation when transitioning to an application based on these results, because the fabrication method is poorly suited for a cost-effective, high-throughput production of surfaces, but higher throughput methods typically come with a necessary compromise on the control of the texture topography.

To overcome this issue, it is necessary to demonstrate the validity of the existing models to LIS with the less regular surface topographies that can be obtained with more easily scalable fabrication techniques. The droplet-assisted growth and shaping (DAGS) method^20−22^ is an ideal candidate for this purpose, because a wide variety of polysilsesquioxane (PSQ) nano- and microstructures, ranging from random networks to well-ordered arrays, can be grown on a surface by tuning the parameters of a reaction between a trichlorosilane precursor and a humid atmosphere. Germanium oxide and aluminum oxide nanostructures can be obtained as well.^23,24^ This process is easily scalable^25^ and has already been successfully used to provide the solid texture component of LIS.^26−29^

In this work, therefore, we will investigate the viscous friction of water droplets on DAGS-derived LIS, focusing on the first few droplets moving along the same path and how they influence the underlying surface. In doing so, we will discuss the existence of a previously unreported memory effect that cannot be explained with a simple lubricant depletion. Our interpretation of this effect on the basis of the smoothing out of an initially conformal lubricant layer suggests that it might be present on any LIS with similar characteristics.

## Experimental Section

### Materials

Trichloro(ethyl)silane (TCES, 98.7%, CAS Registry Number 115-21-9) was purchased from Merck (Germany), and polydimethylsiloxane oil (PDMS, 20cSt, CAS Registry Number 63148-62-9) was purchased from Sigma-Aldrich (St. Louis, MO, U.S.A.). Si wafers (50 mm diameter, B doped, mirror polished) were acquired from CrysTec GmbH (Germany).

### Preparation of Surfaces

#### DAGS Coating of Si Wafers

Si wafers were rinsed with acetone and deionized water and blow-dried with a dry N_2_ stream, repeating the procedure twice. The surfaces were then cleaned in an O_2_ plasma chamber (Femto, Diener Electronics, Germany) for 20 min at 100 W. After this, they were immediately rinsed twice with ultrapure Milli-Q water and blow-dried with dry N_2_. The cleaned samples were introduced in a custom-made glass reaction vessel equipped with a water jacket connected to an external thermostat,^22^ and the temperature *T* was fixed at 23.0 °C ± 0.2 °C, while the relative humidity (RH) was adjusted by flushing the vessel with an appropriate mixture of water-saturated and dry N_2_.^20,21^ After 1 h of flushing, the vessel was sealed and an amount *n*_TCES_ of TCES was injected through a septum; the silane was left to react with the water vapor according to the DAGS mechanism for a given time *t*_r_. The reaction parameters were adjusted as shown in Table 1 to produce two kinds of nanostructures on the surfaces: silicone nanofilaments (SNFs) and rods. The sample was finally subjected to a thermal treatment in air overnight at 200 °C.

**Table 1:** Reaction Conditions Used for the DAGS Coating of Si Wafers

sample	*T* (°C)	RH (%)	*t*_r_ (min)	*n*_TCES_ (mmol)
SNFs	23.0 ± 0.2	45.0 ± 1.5	120	2.25 ± 0.04
rods	23.0 ± 0.2	70.0 ± 1.7	30	2.25 ± 0.04

#### Liquid Infusion

Samples were placed on a custom holder that allowed them to rest at an inclination of 70°. The surfaces were then overfilled by pipetting 10 μL of PDMS oil/cm^2^ of surface, and excess oil was left to drain overnight (16 h). Filter paper (Grade 41, Whatman, U.K.) was placed below the sample holder to collect the drained oil, taking care to avoid direct contact with the samples. The samples were characterized immediately after infusion.

### Characterization

#### Scanning Electron Microscopy (SEM)

Appropriately cut samples were coated with a conductive 12 nm thick Pt layer using a Safematic CCU-010 sputter coater (Safematic GmbH, Switzerland) equipped with a rotating planetary stage. SEM imaging was carried out with a Zeiss GeminiSEM 450 (Carl Zeiss AG, Germany), using an acceleration voltage of 5 kV. Minor and uniform adjustments to image brightness and contrast were performed after acquisition.

#### Contact Angle Goniometry

Water wettability of the surfaces was characterized at 22 °C ± 1 °C using Milli-Q water on a DSA100 contact angle goniometer fitted with a PA3220 tilting device (Krüss GmbH, Germany). Contact angles θ were calculated using the Laplace–Young fitted routing provided by the device software (ADVANCE, Krüss GmbH, Germany) on 10 μL water droplets. The apparent water contact angle θ_app_ of LIS was taken as the tangent to the inflection point of the droplet profile.^30,31^ Sliding angles α_s_ were measured by tilting the sample table at a 0.1 ° s^–1^ speed and recording the angle at which both the advancing and receding front of the droplet started moving. At least nine unique replicate measurements were carried out for each sample. The reported values are the average of these measurements, while uncertainty was taken as one standard deviation.

#### Droplet Dynamics

The dynamics of droplets moving on LIS in different experimental configurations were studied by recording their motion and extracting the droplet position as a function of time from the acquired videos. All experiments were conducted at 22 °C ± 1 °C using ultrapure Milli-Q water on a DSA100 contact angle goniometer fitted with a PA3220 tilting device (Krüss GmbH, Germany) and equipped with a high-speed camera (acA1920-155um, Basler AG, Germany). Sample tilt α and droplet dosing were controlled with the Krüss ADVANCE software, while the camera was controlled with the Basler Pylon Viewer software. An additional, custom-built manual tilting stage was installed on top of the pre-existing stage to add a second axis of rotation. Samples were placed on the tilting table, and a series of water droplets of volume *V* were deposited on the same spot of the tilted surface, thus moving along the same path, with a given time interval *t*_int_ between them. Tilt angle α, droplet volume *V*, and time interval *t*_int_ were varied according to the requirements of the experiment. The velocity and acceleration of droplets were calculated numerically from the acquired signals using a total variation regularization (TVR) approach to minimize noise.^32^ At least three unique replicate measurements on different surface spots were carried out for each sample and each experimental configuration. All reported uncertainties are equal to one standard deviation. The code used for this analysis is available at 10.5281/zenodo.7560309.

## Results and Discussion

### Surface Topography

**Figure 1:**
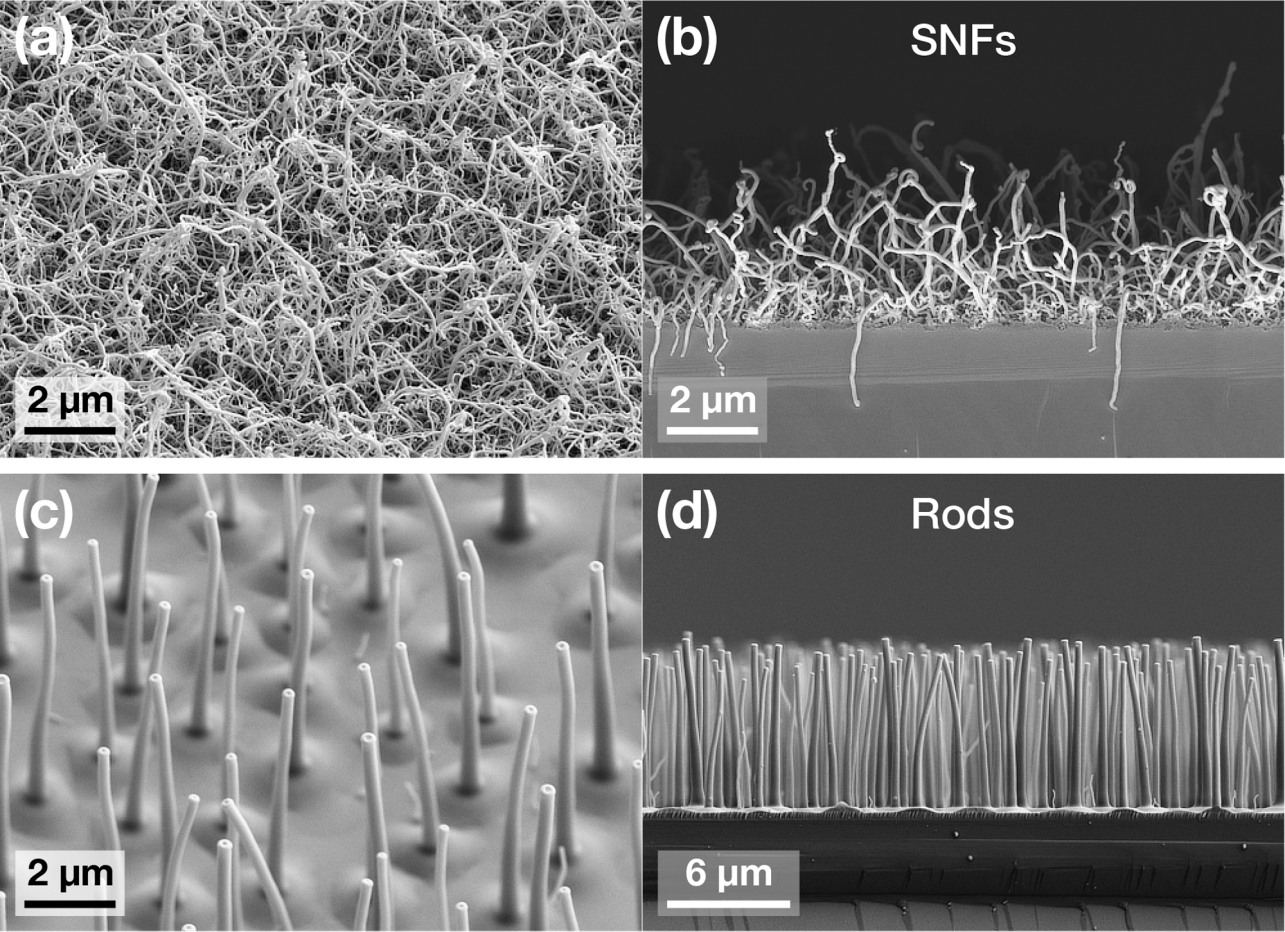
SEM images of the surfaces used for this study: (a and b) SNFs, (a) tilted view and (b) side view; (c and d) rods, (c) tilted view and (d) side view.

The topography of the surfaces used throughout this study is presented in Figure 1. Two vastly different kinds of nanotexture shapes were obtained via the DAGS process: random network silicone nanofilaments (SNFs) and well-ordered rods, shown in panels a and b of Figure 1, respectively. Rods are considerably larger than SNFs and exhibit a higher coating thickness (7.6 μm ± 1.2 μm versus approximately 2 μm); on the other hand, SNFs have a higher texture density ϕ, although a quantitative estimate of this parameter is not trivial as a result of the complex topography of the nanostructures.

The wettability of the tested surfaces, before and after infusion with PDMS oil, is summarized in Table 2 in terms of their water contact angle and sliding angle. On the basis of this characterization, both nanostructures yielded nearly indistinguishable LIS, with little to no observable wetting ridge (see Figure S1 and section S2.1 of the Supporting Information). In particular, the apparent water contact angle of LIS θ_app_ can be compared to its theoretical prediction. In the limit of a vanishingly small wetting ridge, this can be obtained using modified Young’s equation^15,33^
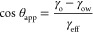
1where γ_o_ is the oil surface tension, γ_ow_ is the oil–water interfacial tension, and γ_eff_ is an effective surface tension whose value reflects the presence or absence of an oil cloaking layer. In our system, PDMS oil cloaks the water droplet, with γ_o_ = 21 mN m^–1^ ^34^ and γ_ow_ = 40 mN m^–1^,^35^ and the spreading parameter ∑_ow_ = γ_w_ – (γ_o_ + γ_ow_) is positive. In this case, γ_eff_ = γ_o_ + γ_ow_, which yields a predicted value for θ_app_ of 108°, in good agreement with the experimental data in Table 2 and previous reports on similar systems.^36^

**Table 2:** Contact Angle θ and Sliding Angle α_s_ of a Water Droplet on Liquid-Infused Surfaces before and after Liquid Infusion

	before infusion		after infusion	
sample	θ (°)	α_s_ (°)	θ_app_ (°)	α_s_ (°)
SNFs	158 ± 2	11 ± 2	108 ± 1	0.5 ± 0.1
rods	169 ± 2	1.0 ± 0.3	107 ± 1	0.3 ± 0.1

Moreover, the absence of a prominent wetting ridge can be taken as an indication that little to no excess lubricant was present on the surfaces.^30,37^

### Friction Scaling

One of the most straightforward ways of experimentally validating a physical model is checking its scaling with respect to its independent variables. In the case of a droplet moving on a LIS at sufficiently low capillary number Ca, the overall viscous dissipation *F*_η_ has the form^8,9,15−17^
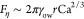
2where *r* is the droplet radius; for this problem, the capillary number can be written as Ca = η_o_*v*_drop_/γ_ow_, with *v*_drop_ as the droplet velocity. As already introduced, droplet friction is composed of a texture-independent term and a texture-dependent term, and the latter implies that the prefactor of eq 2 scales with ϕ^2/3^.^8^

**Figure 2:**
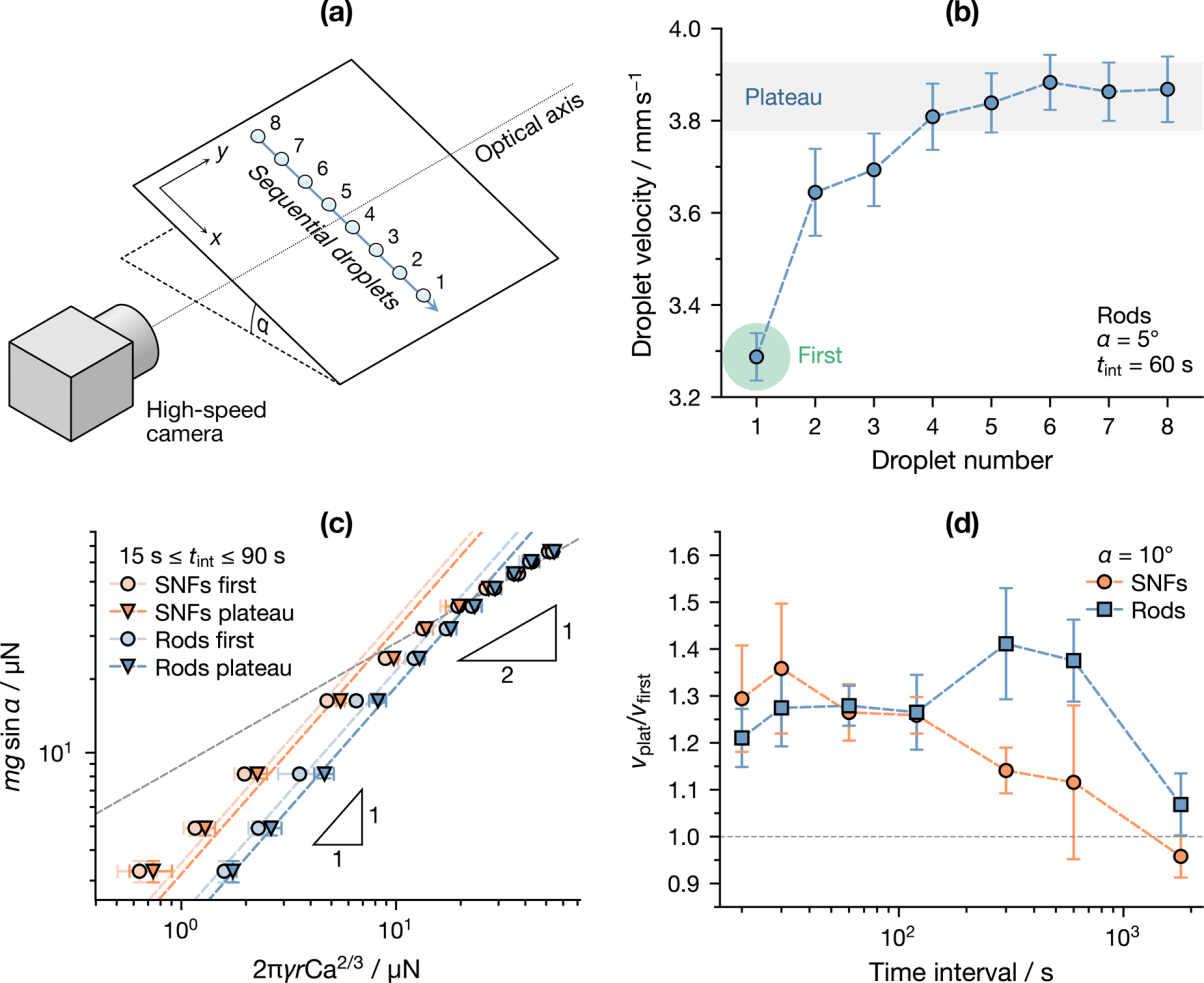
(a) Scheme of the experiment used to determine the scaling of the droplet friction as a function of Ca, (b) detail of a single experiment, showing the plateauing effect observed for successive droplets, (c) driving force as a function of the expected friction scaling, and (d) ratio of the plateau velocity over the first droplet velocity as a function of the time interval between successive droplets.

The relationship between *F*_η_ and Ca can be probed by measuring the equilibrium velocity (see section S1.1 of the Supporting Information) of droplets subject to a constant driving force *F*_d_. In this work, this was accomplished by tilting the surface at an angle α, in a range between 2° and 45°, and recording the motion of a sequence of eight droplets (*V* = 10 μL), as summarized in Figure 2a. While performing the experiments, an unexpected phenomenon was consistently observed: each successive droplet was faster than the previous droplet, until they reached a plateau value after four or five droplets. An example of this is shown in Figure 2b for infused rods tilted at 15°. A description of such a consistent behavior is absent from the literature: as a matter of fact, Keiser et al.^8^ did mention that early droplets might have unusual behavior, but the authors did not allude to any systematic effect nor did they discuss the matter further. The observation of this droplet memory effect prompted us to make the study of its origin the main focus of this work.

To this purpose, the droplet friction scaling for infused SNFs and rods was split between first and plateau droplets, as shown in Figure 2, where the gravitational driving force *mg* sin α was plotted against the expected friction from eq 2. All data appear to follow the predicted friction scaling for sufficiently small Ca, after which they collapse onto a single texture-independent regime with *F*_η_ ∼ Ca^1/3^, appearing with a slope of 1/2 in the figure. This transition was also reported in previous work,^8^ and it has been attributed to the development of a fully dynamic meniscus below the droplet at higher Ca. Because no systematic memory effect was observed in this regime, further analysis will be limited to droplets with low capillary numbers (Ca < 0.005).

Focusing now on the more interesting low-Ca regime, it can be clearly seen that the SNF curves are shifted leftwards to those of rods, implying a higher prefactor of eq 2: this can be easily explained by taking into account the higher ϕ of SNFs. Moreover, the fact that both the first and plateau droplets follow the same scaling suggests that the underlying dissipation mechanism is fundamentally the same, despite the friction experienced by the first droplet being consistently higher.

Because this memory effect is only present in the surface-dependent regime, the most probable cause for the observed behavior seems to be an alteration of the surface caused by the moving droplets, likely in the form a lubricant rearrangement. It is well-known that droplets can, over time, remove lubricant from a LIS.^15^ However, droplets on depleted and partly depleted LIS typically experience an increased friction, up to the point of pinning,^8,16,38^ which is the opposite to what we observed. It seems unlikely, then, that a simple depletion is the only cause of the memory effect: further investigation is therefore in order.

An immediate question that has to be addressed is whether this effect is permanent or not. To determine this, surface tilt was fixed at 10° and the interval between each successive droplet *t*_int_ was varied between 20 s and 30 min; the ratio between the plateau velocity *v*_plat_ and the velocity of the first droplet *v*_first_ was then evaluated as a metric of the friction reduction for consecutive droplets. The results of this experiment are shown in Figure 2d: a clear decreasing trend can be observed for SNFs, with the *v*_plat_/*v*_first_ ratio approaching 1 after waiting 30 min, which implies a return of the surface to its original state. When it comes to rods, on the other hand, the situation is less clear-cut: the *v*_plat_/*v*_first_ ratio appears to initially increase, although it still shows an overall decrease for sufficiently long *t*_int_.

It is apparent, therefore, that a time-dependent relaxation of the surface perturbation indeed exists for both surfaces; moreover, the relaxation behavior appears to be influenced by surface texture, although the exact mechanism is not clear from the experimental results. These considerations further support the hypothesis of a lubricant rearrangement, with the observed texture dependency of relaxation likely caused by capillary effects.

### Droplets Crossing Paths

Despite the evidence presented in the previous section, the position and extent of the surface alteration causing the memory effect as well as its relationship with droplet properties still remain unclear. To address this gap, it is necessary to spatially resolve the perturbation and its influence on droplet friction. Therefore, we devised an experiment with the aim to study how and if the dynamics of the droplets are affected when they cross at 90° the trace left by another sequence of droplets.

**Figure 3:**
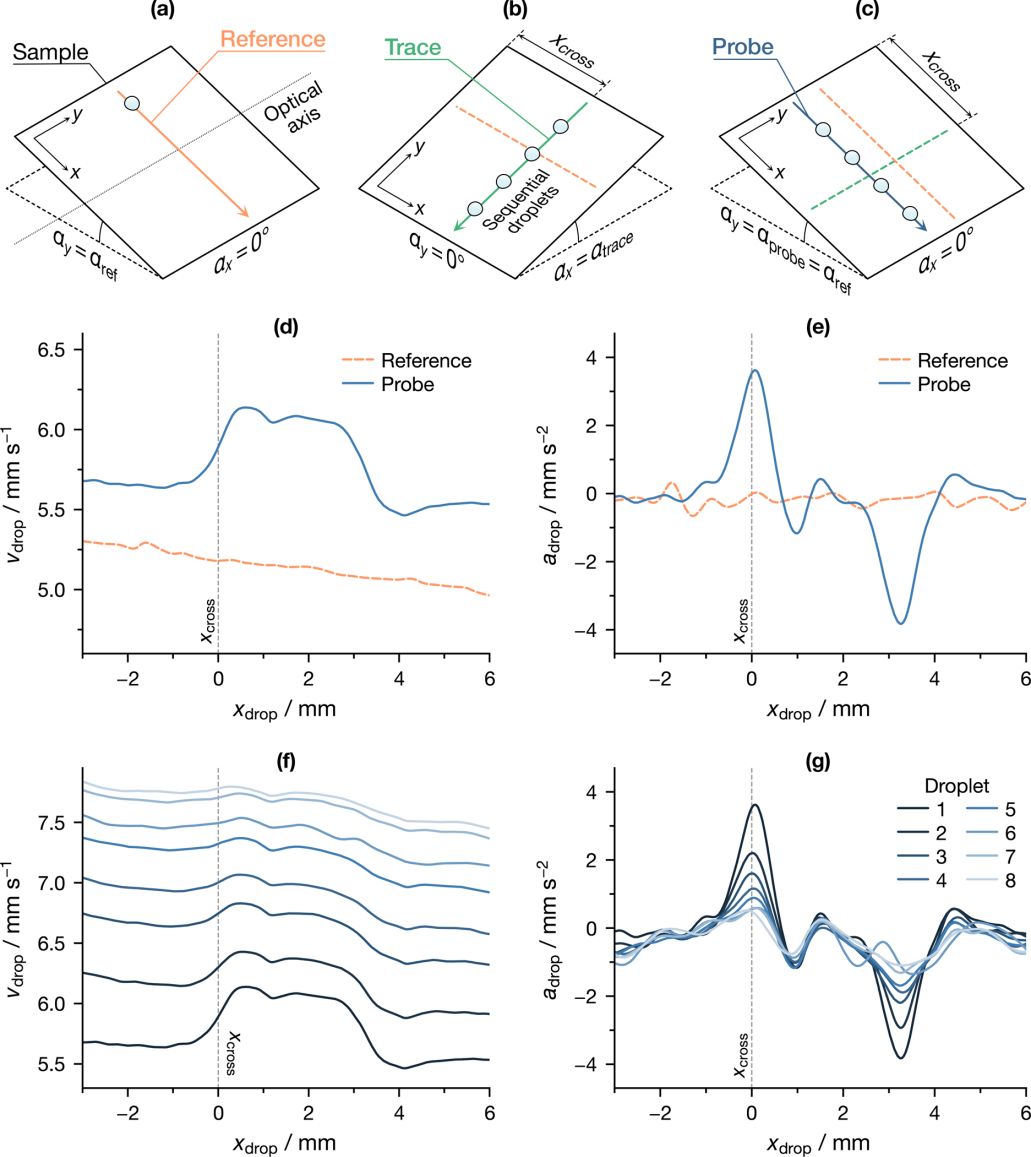
(a–c) Scheme of the droplet crossing experiments, showing its three main steps: (a) reference, (b) trace, and (c) probe droplet motion and (d–g) detail of a single experiment (rods, *V*_trace_ = 15 μL, *V*_probe_ = 10 μL): (d) velocity and (e) acceleration of the reference and first probe droplet and (f) velocity and (g) acceleration of consecutive probe droplets.

A scheme of the experiment is shown in panels a–c of Figure 3, comprising three steps: (1) A single reference droplet of volume *V*_ref_ was left to travel in the *x* direction on a surface tilted at an angle α_ref_. (2) Eight trace droplets of volume *V*_trace_ were deposited at a position *x*_cross_ (see section S1.2 of the Supporting Information) and left to travel in the *y* direction on the surface, tilted at an angle α_trace_. (3) Eight probe droplets of volume *V*_probe_ = *V*_ref_ were deposited on a nearby but separate spot to the reference droplet and left to travel in the *x* direction on the surface, tilted at an angle α_probe_ = α_ref_. The droplets would cross the path traveled by the trace droplets at *x*_cross_.

The motion of probe droplets was then analyzed in the vicinity of the crossing position *x*_cross_, and the influence of probe and trace droplet size was evaluated by varying their volume while keeping Ca constant. Further details are available in section S1.3 of the Supporting Information.

The detailed results of a single experiment are presented in panels d–g of Figure 3. First, the velocity *v*_drop_ of the first probe and reference droplets in the vicinity of the crossing position is shown in Figure 3d. A clear positive peak can be seen in the probe signal, while it is absent from the reference signal: this can be taken as a first indication that the trace droplets created a localized low-friction surface alteration along their path, whose size was roughly comparable to that of the trace droplets themselves (*d*_trace_ = 3.42 mm ± 0.03 mm). An evaluation of this region in terms of the droplet acceleration *a*_drop_, shown in Figure 3d, reveals a global maximum and a global minimum, whose positions can be used to provide a reliable estimate of the velocity peak width. In addition to that, Figure 3c also shows several smaller secondary peaks, highlighting the complexity of the underlying phenomenon. Moreover, droplet acceleration provides a more direct picture of instantaneous friction than *v*_drop_, because a non-zero acceleration implies a mismatch between actual friction and its local equilibrium value, being the driving force constant.

The evolution of probe velocity and acceleration as more and more droplets crossed the trace by traveling along the same path is shown in panels f and g of Figure 3. Two main features can be noticed in the velocity signals (Figure 3f): first, that the average *v*_drop_ increased with each consecutive droplet, as already discussed in the previous section; second, that the trace peak was gradually smoothed over. The latter is also evident from the acceleration signals (Figure 3g), as the global maximum and minimum were progressively flattened, although it did not disappear completely. This can be interpreted as the droplets “matching” or possibly altering the pre-existing surface perturbation. Nevertheless, a memory of the latter was partly retained, at least within the limits of our experiment.

**Figure 4:**
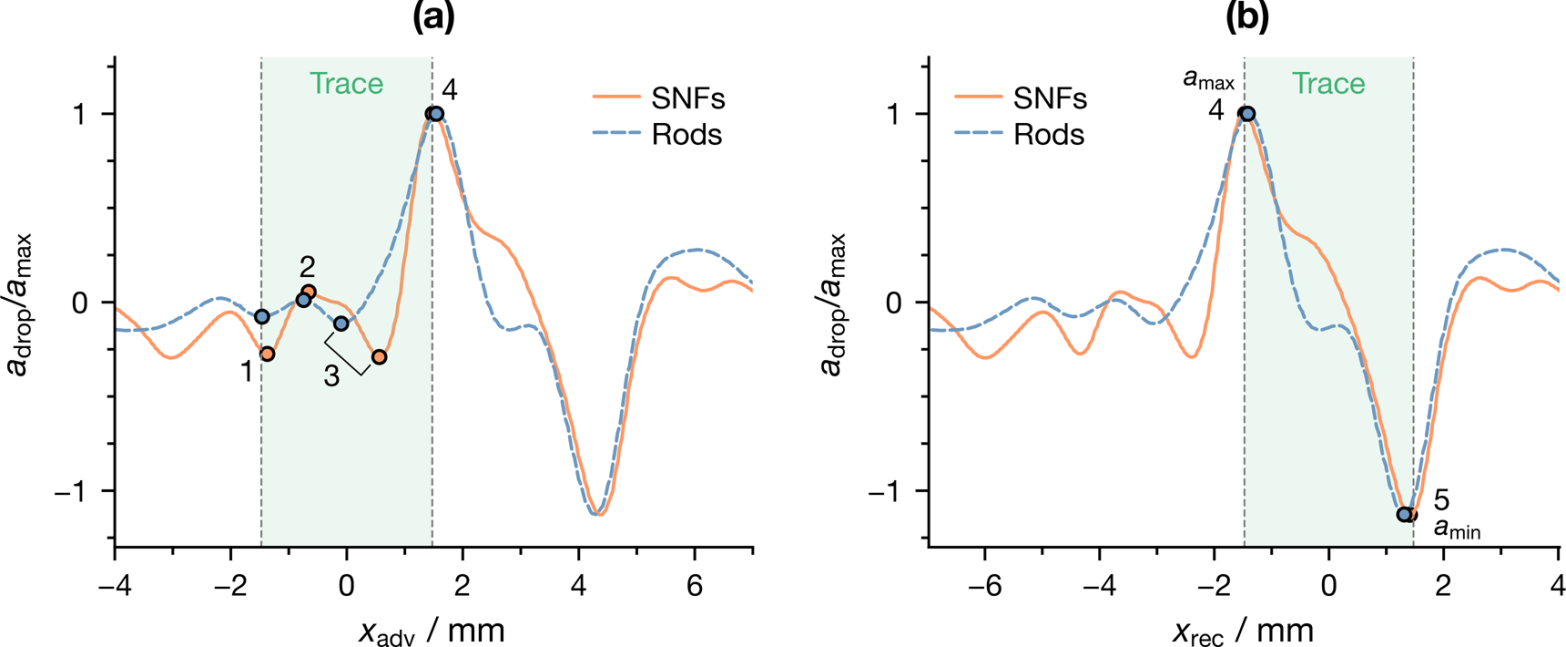
Normalized acceleration of the first probe droplet (*V*_probe_ = 10 μL) crossing a trace (*V*_trace_ = 10 μL) as a function of the (a) advancing and (b) receding front position. The shaded areas represent the nominal extent and position of the trace.

Up to now, droplet crossing has only been discussed in terms of the overall droplet position, that is, the position of the droplet center of mass. However, the overall friction felt by the droplet is a sum of all of the contributions from the surface below it: the droplet should then, in principle, start being influenced by the trace as soon as its advancing front enters it and stop when its receding front leaves it. To verify this assumption, Figure 4 shows the acceleration of the first probe droplet of a representative experiment on infused SNFs and rods as a function of its advancing (*x*_adv_) and receding (*x*_rec_) front position; the nominal position of the trace, assumed to be equal to the trace droplet base diameter and centered on the trajectory of its center of mass, is indicated as a shaded area in Figure 4. Acceleration was normalized by its maximum value to facilitate the comparison between different surfaces.

It can be seen from the figure that both surfaces had a very similar behavior. As the advancing front enters the trace (Figure 4a), three small peaks (1, 2, and 3) can be observed: while separating these peaks from the noisy baseline is not always straightforward, their rising and falling is nevertheless an indication of a competition between a low and a high friction zone that were trying to speed up and slow down the droplet, respectively. As a larger portion of the droplet was inside the trace, a clear global maximum of acceleration *a*_max_ (peak 4) was reached: this occurred when the receding edge touched the trace (i.e., when the whole droplet was inside the trace). Acceleration then decreased, switching its sign around the time half of the droplet was outside the trace, before reaching a minimum *a*_min_, i.e., maximum deceleration (peak 5), when the receding edge touched the outer border of the trace and the droplet was fully outside it.

As already stated, non-zero acceleration implies a mismatch between the instantaneous viscous friction and the constant driving force. Therefore, *a*_max_ corresponds to the minimum friction experienced by the droplet; vice versa, negative *a*_min_ corresponds to the maximum friction. In light of this consideration, then, the remarkable match between the positions of *a*_max_ and *a*_max_ and the instants when the droplet had fully entered and fully left the trace, respectively, clearly proves that droplets created a low-friction region as they moved on the tested LIS. This should be intended in the sense that the surface properties in the trace are such that, at equilibrium, they would result in a higher droplet velocity for the same driving force.

**Figure 5:**
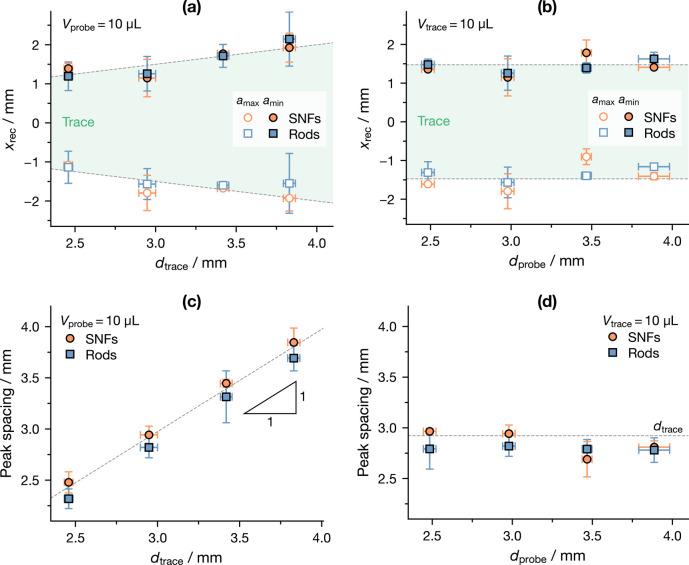
Position of the global maximum and minimum of acceleration in terms of the (a and b) receding front and (c and d) their spacing as a function of the base diameter of (a and c) trace *d*_trace_ and (b and d) probe *d*_probe_ droplets. The shaded areas represent the nominal extent and position of the trace.

The evidence presented thus far seems to suggest that the width of the region altered by the trace droplets is very close to their base diameter *d*_trace_. It is therefore useful to study the position of *a*_max_ and *a*_min_ for trace and probe droplets of different sizes; on the basis of the results shown in Figure 4b, the position of the receding front *x*_rec_ appears to be the most suited for this purpose. Panels a and b of Figure 5 show such an analysis as a function of *d*_trace_ and *d*_probe_, respectively: it can be clearly seen that what is already observed in Figure 3 can be extended to droplets of different sizes, because the position of *a*_max_ closely matches the receding front of the probe entering the trace, while *a*_min_ matches the receding front leaving it. Moreover, these positions do not seem to be influenced by the size of the probe droplet, as shown in Figure 5b.

It is interesting then to evaluate the peak spacing, that is, the difference between the position of *a*_min_ and *a*_max_, as a function of *d*_trace_ and *d*_probe_, as shown in panels c and d of Figure 5. As expected, the peak spacing almost exactly matches *d*_trace_: this is particularly evident in Figure 5d, where the two quantities are shown to have 1:1 scaling (−0.02 mm ± 0.02 mm intercept; adjusted *R*^2^ = 0.978). It is therefore clear that not only do droplets alter the LIS on which they travel by creating a low-friction zone but this alteration is as wide as the droplets themselves, as initially supposed from Figure 3d.

### Physical Interpretation of the Memory Effect

It seems highly likely that the alteration observed in droplet crossing experiments is the same as the alteration postulated as the origin of the memory effect for scaling experiments.

This problem is strikingly reminiscent of the adaptive wetting of solid surfaces.^39,40^ As a matter of fact, it has been reported that solid surfaces can adapt to the liquid that they are in contact with through reversible molecular reorganization at the solid–liquid interface, generally leading to a more favorable interaction. LIS, however, are characterized by the presence of a “free” lubricant layer and a liquid wetting ridge: this is different from adaptive PDMS organogels, a system that otherwise bears a strong resemblance to LIS.^41^ Unlike organogels, where unreacted oligomers can migrate from the bulk to the surface, causing a wetting-induced lubrication, LIS are already in such a state. This leaves the reorientation of liquid molecules at the lubricant–water interface as a possible adaptive mechanism. The time scale for this can be estimated on the basis of the time that a liquid molecule takes to diffuse its own diameter:^39^ for the range of properties of our lubricant, it is approximately 20 ns (section S2.2 of the Supporting Information). This does not seem compatible with our findings of comparatively long (>10^3^ s) relaxation times.

These considerations, together with the microscale displacements caused by the moving oil menisci, suggest that the memory effect that we observed has its origin in the unique features of LIS. We can then finally attempt to propose a physical interpretation for this phenomenon, although it should be kept in mind that, in the absence of a direct visualization of the lubricant layer, this interpretation is necessarily speculative.

**Figure 6:**
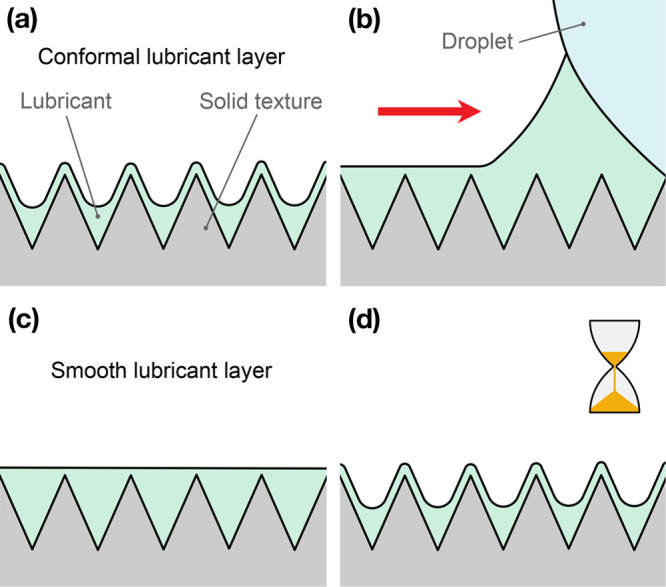
Scheme of the proposed mechanism for the droplet memory effect: (a) lubricant is initially conformal to the surface texture; (b) droplet deposits a thin lubricant at its back; (c) LIS surface is temporarily smoothed; and (d) after some time, the LIS returns to its original configuration.

As previously discussed, because a lubricant depletion would have led to a friction increase, the trace was likely not a depleted region; instead, if this assumption were true, moving droplets would have caused a lubricant enrichment in their wake, at least in the tested limit of a comparatively small number of droplets on fresh surfaces. Despite the appearances, this is not incompatible with previous theoretical models and experimental evidence, although the key lies in the initial configuration of the surface. As a matter of fact, LIS with a nano- or microtextured liquid interface can be obtained when a thin lubricant layer conforms to the underlying surface texture,^6,12,38,42^ as shown in Figure 6a; droplets moving on such surfaces typically experience a higher friction than on smoother LIS.^38^ From the point of view of the model encapsulated by eq 2, we can interpret this observation with a higher effective ϕ. However, as already introduced, a thin lubricant film is deposited at the back of the moving droplet according to the LLD model (Figure 6b), whose height is at least equal to the texture height:^16,43^ this might result in a temporary smoothing out in full or in part of the conformal lubricant layer (Figure 6c), leading to a friction reduction when compared to the original configuration. This alteration would be clearly confined to the region of the surface touched by the moving droplet,^15^ as observed in droplet crossing experiments; we can also rationalize the gradual plateauing of droplet velocity shown in Figure 2b as the progressive accumulation of such an effect. Finally, because, for these LIS, a smooth lubricant layer is not the stable configuration, oil would gradually diffuse back into the texture (Figure 6d): this would then explain the time- and texture-dependent relaxation behavior shown in Figure 2d.

It is reasonable to expect, however, that, for a sufficiently large number of droplets traveling along the same path, a depletion-induced friction increase will eventually dominate.

## Conclusion

The findings presented in this work can be summarized in two main points. First, the existing friction laws for droplets on LIS also hold true for surfaces with random or semi-regular solid texture, which further confirms their generality. This facilitates the description of surfaces produced with cost-effective high-throughput techniques, such as the DAGS method, that typically offer lower geometrical control than lithographic procedures. Second, a droplet memory effect can exist on LIS, where moving droplets create a low-friction region in their wake and influence the motion of future droplets. This effect follows a time- and texture-dependent relaxation, and it appears to be largely unrelated to lubricant depletion.

The observed memory effect is of particular importance for practical applications: it can be a beneficial effect, because droplets are able to create preferential low-friction pathways on surfaces, or it can be detrimental, because not all droplets will move with the same velocity. Dependent upon the use case, therefore, it should either be suppressed or enhanced, but its possible presence should always be taken into account when designing a LIS.

We attributed this effect to the initial configuration of the lubricant layer on the tested surfaces, where the lubricant is possibly conforming to the underlying texture and creates a nanorough liquid surface. The lubricant layer can then be temporarily smoothed-out by moving droplets, owing to a Landau–Levich–Derjaguin thin-film deposition at the receding oil menisci, causing the observed friction reduction.

Future work should therefore aim at the development of a quantitative model for this phenomenon, a goal that would be greatly aided by the direct visualization of the altered low-friction region, for example, by means of confocal microscopy or interferometric techniques.

## Supporting Information Available

Additional experimental details: constant velocity validation, determination of the crossing position, and droplet size in crossing experiments and supplementary results: apparent water contact angle on LIS and reorientation of liquid molecules (PDF)
