# Supplementary material for: Droplet Memory on Liquid-Infused Surfaces
Source: Langmuir. 2023 Apr 17;39(17):6160–8. doi: 10.1021/acs.langmuir.3c00289 (PMC10157887; doi:10.1021/acs.langmuir.3c00289)
Supplement: Supplementary file 1 — la3c00289_si_001.pdf [file la3c00289_si_001.pdf]

Droplet memory on liquid infused surfaces

Davide Bottone and Stefan Seeger*

University of Zurich, Department of Chemistry, Winterthurerstrasse 190, 8057, Zurich, Switzerland

E-mail: sseger@chem.uzh.ch

Supporting Information

S1 Additional experimental details

S1.1 Constant velocity validation

The study of droplet friction scaling relies on the assumption of an equilibrium between viscous dissipation and gravitational driving force, which results in a constant droplet velocity.

Therefore, only sufficiently constant velocity signals were considered for this analysis. This was validated by verifying that the average of the relative velocity gradient along the x axis did not exceed 1 % for at least 6 mm, that is:

$$\frac{\partial v(x)}{\partial x}\frac{1}{v(x)} \leq 0.01\,. \tag{S1}$$

Moreover, even if Equation S1 was satisfied, the maximum allowed standard deviation of velocity was 4 % of its average value. Velocity signals that did not meet these conditions were excluded from this analysis.

Table S1: Values of V and α yielding the same Ca.

V / µL	α / °
6	14.1
10	10
15	7.6
20	6.3

S1.2 Determination of crossing position

The crossing position x_{cross} was chosen at least 20 mm from the deposition position of reference and probe droplets. Moreover, it was ensured that the motion of reference and probe droplets was recorded for at least 10 mm before they encountered the crossing point, in order to clearly observe their behavior at x_{cross}.

The exact position of x_{cross}, as well as the base diameter of the trace droplets d_{trace}, was extracted from still images of the trace droplets, as their motion was parallel to the optical axis of the camera.

S1.3 Droplet size in crossing experiments

The influence of probe and trace droplet size in crossing experiments was evaluated by varying their volume between 6 µL and 20 µL, and surface tilt was changed accordingly to maintain the same Ca. This was accomplished by noting that, for any two droplets 1 and 2, if Equation 2 is valid:

$$\alpha_2 = \arcsin\left(\sin\alpha_1\left(\frac{V_1}{V_2}\right)^{2/3}\right). \tag{S2}$$

In our experiments, $\alpha_1 = 10°$ and $V_1 = 10$ µL, and the values of V and α employed are summarized in Table S1. When setting α_{trace}, the values in Table S1 were rounded to the nearest integer, owing to the lower resolution of the custom tilting stage scale.

S2 Supplementary results

S2.1 Apparent water contact angle on LIS

The definition of an apparent contact angle on LIS θ_{app} is not trivial,[1] owing to the presence of a lubricant wetting ridge around the test liquid droplet.

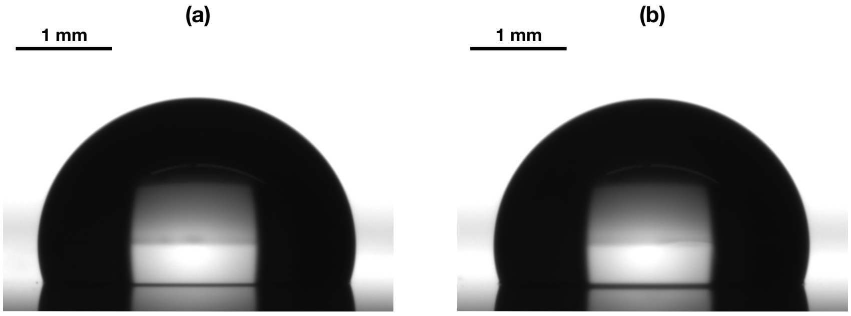

Figure S1: Water droplets ($V = 10\,\mu$L) on liquid infused SNFs (a) and rods (b).

Table S2: Values of water contact angle on LIS calculated with the extrapolation and inflection point methods.

	θ_{app} / °	
**Sample**	**Extrapolation**	**Inflection point**
SNFs	109±1	108±1
Rods	108±1	107±1

Two main methods have been discussed in the literature. The first is based on the extrapolation of the droplet spherical cap to the substrate profile, neglecting the presence of the wetting ridge.[2] The second, instead, relies on the calculation of the tangent angle at the inflection point of the droplet profile.[3,4] The two methods coincide in the case of a vanishingly small wetting ridge.[1]

In our LIS, we did not observe prominent wetting ridges during water contact angle measurements, as shown in Figure S1. For the sake of completeness, we calculated θ_{app} with both of the aforementioned methods, as summarized in Table S2. The analysis was performed with the Krüss ADVANCE software, using the Laplace–Young fitting routine for the extrapolation method and the Tangent routine for the inflection point method. Results obtained with the two methods are in fairly good agreement, as expected for small wetting ridges. In the main text, we discuss only the values obtained with the inflection point method, due to their more straightforward physical interpretation.[4]

S2.2 Reorientation of liquid molecules

The time required for liquid molecules to change their orientation at a given temperature T can be estimated from the time required for the molecules to diffuse their own diameter:[5]

$$\tau = \frac{\pi\eta M_W}{2N_A\rho k_B T}, \tag{S3}$$

where M_W is the molecular weight, ρ is the density, N_A is the Avogadro constant, and k_B is the Boltzmann constant.

For the PDMS oil we used in our experiments, $\eta = 19\,\mathrm{mPa\,s}$ and $\rho = 0.95\,\mathrm{g\,cm^{-3}}$. We can estimate the molecular weight from interpolation of literature data on M_W as a function of kinematic viscosity,[6] obtaining $M_W \approx 1800\,\mathrm{g\,mol^{-1}}$. Equation S3 then yields a reorientation time $\tau \approx 23\,\mathrm{ns}$ at 22 °C.

References

(1) McHale, G.; Afify, N.; Armstrong, S.; Wells, G. G.; Ledesma-Aguilar, R. *Langmuir* **2022**, *38*, 10032–10042.

(2) Guan, J. H.; Wells, G. G.; Xu, B.; McHale, G.; Wood, D.; Martin, J.; Stuart-Cole, S. *Langmuir* **2015**, *31*, 11781–11789.

(3) Semprebon, C.; McHale, G.; Kusumaatmaja, H. *Soft Matter* **2017**, *13*, 101–110.

(4) Semprebon, C.; Sadullah, M. S.; McHale, G.; Kusumaatmaja, H. *Soft Matter* **2021**, *17*, 9553–9559.

(5) Butt, H.-J.; Berger, R.; Steffen, W.; Vollmer, D.; Weber, S. A. L. *Langmuir* **2018**, *34*, 11292–11304.

(6) Mark, J. E., *Polymer Data Handbook*; Oxford University Press: 1999.
